# A Multi-Sensor Comparative Analysis on the Suitability of Generated DEM from Sentinel-1 SAR Interferometry Using Statistical and Hydrological Models

**DOI:** 10.3390/s20247214

**Published:** 2020-12-16

**Authors:** Ayub Mohammadi, Sadra Karimzadeh, Shazad Jamal Jalal, Khalil Valizadeh Kamran, Himan Shahabi, Saeid Homayouni, Nadhir Al-Ansari

**Affiliations:** 1 Department of Remote Sensing and GIS, University of Tabriz, Tabriz 5166616471, Iran; mohammadi.ayub@tabrizu.ac.ir (A.M.); sa.karimzadeh@tabrizu.ac.ir (S.K.); valizadeh@tabrizu.ac.ir (K.V.K.); 2 Institute of Environment, University of Tabriz, Tabriz 5166616471, Iran; 3 Department of Architecture and Building Engineering, Tokyo Institute of Technology, Yokohama 226-8502, Japan; 4 College of Engineering, University of Sulaimani, Sulaimani 46001, Iraq; shazad.jalal@univsul.edu.iq; 5 Faculty of Built Environment and Surveying, Universiti Teknologi Malaysia, Johor Bahru 81310, Malaysia; 6 Department of Geomorphology, Faculty of Natural Resources, University of Kurdistan, Sanandaj 6617715175, Iran; 7 Department of Zrebar Lake Environmental Research, Kurdistan Studies Institute, University of Kurdistan, Sanandaj 6617715175, Iran; 8 Centre Eau Terre Environnement, Institute National de la Recherche Scientifique, Quebec, QC G1K 9A9, Canada; saeid.homayouni@ete.inrs.ca; 9 Department of Civil, Environmental and Natural Resources Engineering, Lulea University of Technology, 971 87 Lulea, Sweden

**Keywords:** DEM, remote sensing, SAR imagery, Malaysia, Iran

## Abstract

Digital elevation model (DEM) plays a vital role in hydrological modelling and environmental studies. Many essential layers can be extracted from this land surface information, including slope, aspect, rivers, and curvature. Therefore, DEM quality and accuracy will affect the extracted features and the whole process of modeling. Despite freely available DEMs from various sources, many researchers generate this information for their areas from various observations. Sentinal-1 synthetic aperture radar (SAR) images are among the best Earth observations for DEM generation thanks to their availabilities, high-resolution, and C-band sensitivity to surface structure. This paper presents a comparative study, from a hydrological point of view, on the quality and reliability of the DEMs generated from Sentinel-1 data and DEMs from other sources such as AIRSAR, ALOS-PALSAR, TanDEM-X, and SRTM. To this end, pair of Sentinel-1 data were acquired and processed using the SAR interferometry technique to produce a DEM for two different study areas of a part of the Cameron Highlands, Pahang, Malaysia, a part of Sanandaj, Iran. Based on the estimated linear regression and standard errors, generating DEM from Sentinel-1 did not yield promising results. The river streams for all DEMs were extracted using geospatial analysis tool in a geographic information system (GIS) environment. The results indicated that because of the higher spatial resolution (compared to SRTM and TanDEM-X), more stream orders were delineated from AIRSAR and Sentinel-1 DEMs. Due to the shorter perpendicular baseline, the phase decorrelation in the created DEM resulted in a lot of noise. At the same time, results from ground control points (GCPs) showed that the created DEM from Sentinel-1 is not promising. Therefore, other DEMs’ performance, such as 90-meters’ TanDEM-X and 30-meters’ SRTM, are better than Sentinel-1 DEM (with a better spatial resolution).

## 1. Introduction

Digital elevation model (DEM) is one of the essential geospatial data tools used in geological, geographical, geomorphological, and environmental applications. It allows extracting essential terrain features such as slope, aspect, elevation, and many more [1,2,3,4]. The accuracy of such features is directly associated with the accuracy of DEM. There are various geospatial technologies with different technical complexity, geographical coverage, geometric and geographic accuracy, cost, user-effort, and time requirements for generating DEMs. Ground surveying, photogrammetry, light detection and ranging (LiDAR) systems, satellite optical imagery, and SAR interferometry are the best operational technologies that have been used to generate the DEMs for different applications. Compared with the remote sensing (RS) techniques, ground surveying is a more accurate method to create a DEM [5,6]. However, RS-based technologies are relatively less expensive timely and can cover larger areas shortly [7].

Among the RS sensor technologies, SAR Interferometry has shown its advantages for DEM generation, such as sensitivity of SAR signal to the surface physical and topographic properties, independence of weather and illumination conditions, and robust and accurate analytical approaches [8,9]. With the open data strategy by the national space and Earth observation agencies, both scientific and commercial end-users have had access to high spatial and temporal SAR imagery from various wavelengths, polarization, and orbits. Thus, remote sensing has received lots of attention in the last decade [7,10].

Because of the different product types and sensor modes, Sentinel-1 has many practical applications such as subsidence, landslide and, land cover flood detection [11,12,13]. Simultaneously, many researchers have been applying Sentinel-1 for DEM creation (because of freely availability) [14]. Since this study, for the first time, tries to test and compare the accuracy and validity of a derived DEM from Sentinel-1 data with the other DEMs, it can be significant. Therefore, this study opens the researchers’ eyes towards the DEM generation from Sentinel-1 satellite data.

A DEM can be acquired from different sources, including ground-based surveys [15,16,17,18], basic topographic maps [19,20,21,22,23,24,25] and remote sensing technique [8,26,27,28,29,30,31,32,33,34]. Many studies have been simultaneously conducted with the DEM generation by using interferometry synthetic aperture radar (InSAR) technique [9,27,35,36,37,38,39,40,41,42,43,44].

As a very successful example, many researchers worldwide tend to create DEMs from Sentinel-1 SAR data. Braun [45] created a DEM from Sentinel-1 data conducted with the European Space Agency (ESA); since then, it has been a key source for researchers worldwide, but there is no comparative study yet to test its accuracy and validity the other products, especially freely available DEMs. For generating a reliable DEM, the perpendicular baseline (PB) of SAR image data should be between 150 to 300 m [46], while in the best condition, the PB of Sentinel-1 cannot exceed 150 m. Therefore, this can be a significant problem. For this reason, the researchers should be careful about using Sentinel-1 to generate DEM. It is worth mentioning that the other presented DEMs’ capability and applicability are not in the scope of the current study and are just used to test the accuracy of the created DEM.

The main objective of the current study was to generate a DEM from Sentinel-1 satellite imagery by using the InSAR technique and also to compare it with DEMs of airborne synthetic aperture Radar (AIRSAR) product (with 10 × 10 cell size), advanced land observing satellite phase array L-band synthetic aperture radar (ALOS-PALSAR) imagery (with 12.5 × 12.5 m spatial resolution), TanDEM-X (with 90 × 90 cell size) and SRTM (with 30 × 30 cell size) for two different study areas.

## 2. Description of the Study Area

**Figure 1:**
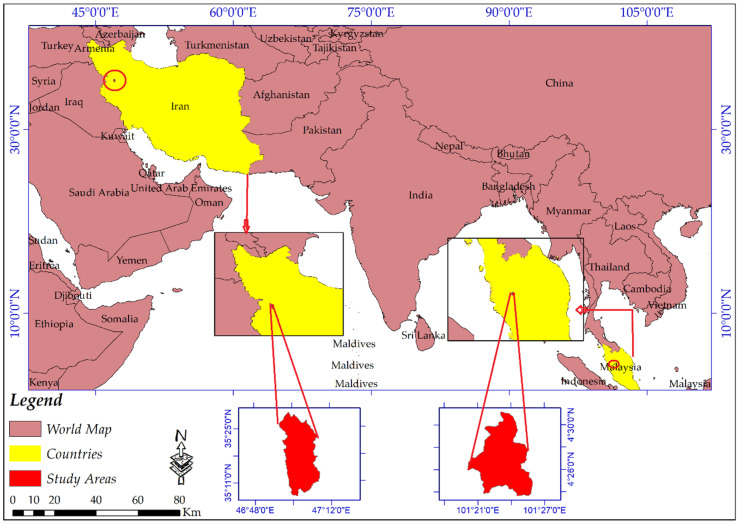
The geographical extent of the study areas.

When a comparative study is done, every aspect, even the study area’s selection, must be considered. For example, regarding SAR data and DEM generation, collecting data on hot days and dense vegetation coverage (especially Band-C) result in low coherence extraction. In this study, two study areas of a part of the Cameron Highlands, Pahang, Malaysia, and Sanandaj county, Kurdistan, Iran, were selected to carry out this comparative study. The Cameron Highlands is mostly covered by dense and sparse vegetation, while vegetation coverage for Sanandaj county is negligible. The Cameron Highlands is located between Longitudes 101°20′00″ to 101°27′10″ East and Latitudes 4°23′30″ to 4°31′10″ North (Figure 1). This scope covers an area of 81.249 km^2^ and is placed in Peninsular Malaysia’s central part and the main range of geological units of Malaysia [47,48]. There are two prominent outcrops in the Cameron Highlands, the first one is acid intrusive (undifferentiated), which covers most of the study area of 60.99 km^2^, and the second formation (Silurian-Ordovician) consists of slate, schist, limestone with minor intercalation of sandstone, and phyllite [49]. Based on the tropical rainfall measuring mission (TRMM) data, the Cameron Highlands’ annual rainfall fluctuates between 3800 mm to 4200 mm annually [49,50].

With a population of almost 432 thousand, the city of Sanandaj is the biggest in Kurdistan Province, Iran. Sanandaj is located in the Longitudes 46°24′00″ to 47°19′00″ East and Latitudes 35°3′00″ to 35°39′00″ North (Figure 1). The city has a cold and semi-arid climate but moderate weather during spring. The maximum and the minimum temperature fluctuate between 44 °C and −13.5 °C. In this study area, vegetation coverages are mostly grass, while trees and high vegetation coverage are less marked. Therefore, the two study areas have different geomorphologies and are appreciated for this comparative study.

## 3. Material and Methods

### 3.1. Image Selection for InSAR DEM Generation

Sentinel-1 satellite data are the first program by Copernicus, conducted by the ESA. Sentinel-1A and Sentinel-1B are two platforms for it [51]. This product has a 5.7 cm wavelength in C-band [12,52]. Crisis management, natural hazards and interferometry studies are among the most applications of the Sentinel-1 mission [53]. Interferometric Wide swath (IW), strip map (SM), extra-wide swath (EW) and wave (WV) are four sensor modes for the Sentinel-1 mission [51]. Single look complex (SLC), ground range detected (GRD), and ocean (OCN) are three types of data products for Sentinel-1 [12,52]. SAR interferometry needs the combination of two images from the same scene but at different times and sensor position [54]. It is worth mentioning that for Sentinel-1 if one wants to collect data with more PB, the TB will dramatically go higher. Table 1 shows the details of the data used in this study.

### 3.2. Pre-Processing and Processing of Data Used

**Figure 2:**
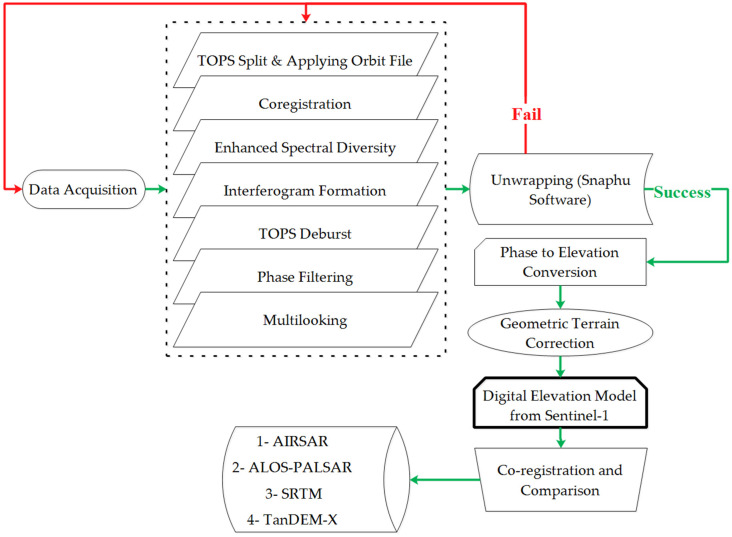
An overview of the proposed methodology.

InSAR technique is the comparison of the phase values for a pair of SAR images [49,55]. The phase information of two or more SAR imageries, acquired over the same region (same geographical position), either simultaneously or at different times, due to the scattered echoes’ monochromatic nature, can be combined [3]. Figure 2 details the DEM creation’s comprehensive methodology from Sentinel-1 satellite imagery and its comparison with the other products.

#### 3.2.1. TOPS Split and Applying Orbit File

**Figure 3:**
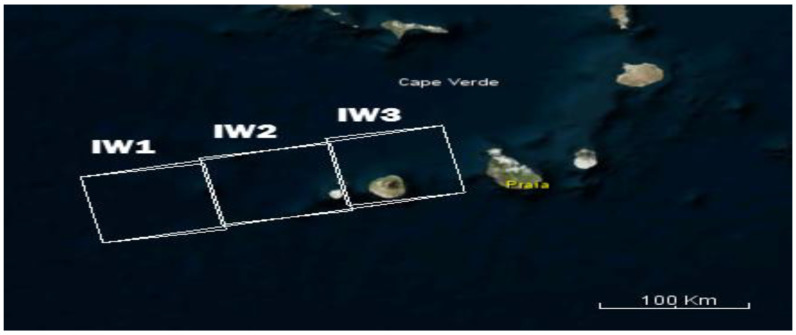
Sub-swaths Interferometric Wide (IW)1, IW2, and IW3 [56].

SLC product has 3 Sub-Swaths of IW1, IW2, and IW3, which each sub swath is for an adjacent acquisition by the TOPS mode (Figure 3). Sentinel-1 TOPS Split is used to select only those bursts which are needed for the analysis. Currently, each Sub-Swath should be processed separately, unless they must be merged. Orbit auxiliary data consists of information related to the satellite’s position during data acquisition [55]. This information should be added to the images’ metadata using the application orbit file operator.

#### 3.2.2. Co-Registration and Enhanced Spectral Diversity

The back geocoding (co-registration) operator in SNAP software was used to co-register the two split satellite data based on the orbital information. In order to extract the phase difference, two acquisitions must be stacked first. This is an essential step in interferogram formation, as it ensures that each ground object has the same range and azimuth pixel in either image [8,46]. Enhanced spectral diversity (ESD) can be employed to increase the quality of the co-registration process. ESD corrects range and azimuth’s shift and is only applied for more than one burst [55], which results in a better co-registered image.

#### 3.2.3. Interferogram Formation and Coherence Estimation

An interferogram is calculated by cross multiplication of the master and slave images [57]. Here, the phase band shows the difference of a pixel in two images [39,40,46]. An interferogram ranging from -π to +π and is displayed in a rainbow color scale, which has variations from topography and surface deformation [57]. On the other hand, these patterns are called fringes and a full 2π cycle, where each cycle is demonstrating half of the sensor’s wavelength [45,57].

The interferogram consists of phase variation ϕ from contributing factors, the Earth’s curvature (phase of flat-earth) ϕflat, the topographic surface (phase of topographic) ϕDEM, conditions of the atmosphere (e.g., pressure change, temperature, and humidity between the two acquisitions) ϕatm, and noise ϕnoise (e.g., change of the scatters, volume scattering and different look angle), and finally the surface deformation ϕdisp (Equation (1)).

(1) ϕ=ϕDEM+ϕflat+ϕdisp+ϕatm+ϕnoise

Interferogram formation represents a raw DEM resolution $\Omega_{r}\;$via an independent number of looks used in the processing (Equation (2)) [31]:

(2) $$\Omega_{r}=\frac{n_{az}\delta_{az}^{gr}+n_{rg}\delta_{rg}^{gr}}{2}$$

where $n_{az}$ and $n_{rg}$ are azimuth and range independent number of looks, respectively, while $\delta_{az}^{gr}$ and $\delta_{rg}^{gr}$ are single SAR pixel azimuth and range ground resolution, respectively.

Coherence is a useful source to know about the quality of an interferogram [57]. Coherence is formed as a separate raster, which shows how similar a pixel can be between the two images ranging from 0 to 1 [57]. Areas of high and low coherence will appear in a bright and dark color, respectively.

#### 3.2.4. TOPS Debursting

TOPS data has 9 bursts, which is typically separated by some specific zones [51]. However, any values within these zones are defined as invalid data and must be debursted. To remove the lines between the bursts, the Sentinel-1 TOPS Debursting operator using SNAP software should be applied to the interferogram.

#### 3.2.5. Phase Filtering and Multilooking

For a proper unwrapping process, phase filtering and multi-looking are two essential steps. Noise from geometric and temporal decorrelation and volume scattering can corrupt the interferometric phase [46,57]. Restoring phase information in decorrelated areas is quite hard, but applying proved phase filters (e.g., the Goldstein filter), could enhance the fringes’ quality in the interferogram [57]. It is a preprocessing technique that considerably enhances the phase unwrapping accuracy and decreases the residues in the phase unwrapping step. The method was first proposed by Goldstein and Werner [58]. For Goldstein filter, adaptive filter exponent was 1 (its value lies in range of 0 and 1, which the larger the value the stronger the filtering process will be), for fast Fourier transformation (window length size of 64) and windows size (size of 3) were remained as default. It is worth mentioning that the coherence mask was not applied.

#### 3.2.6. Phase Unwrapping

Unwrapping was applied in this study by using SNAPHU software [59]. Phase unwrapping (PU) is associated with the convergence of the phase difference from point to point by adding the integer number of cycles, which minimizes the phase difference [8]. PU recovers the integer number of cycles n to be applied to the wrapped phase φ. Therefore, the unambiguous phase value *ψ* can be eventually obtained for each pixel (Equation (3)):

(3) ψ=ϕ+2π.n.

The interferometric phase is somewhat ambiguous and within the scale of 2π can be known only [57]. In order to relate the phase to the topographic height, it must be unwrapped first. PU solves the ambiguity through integrating phase differences among neighboring pixels [57].

#### 3.2.7. Phase to Elevation Conversion and Terrain Correction (TC)

The unwrapped phase is not a metric measure yet. To convert the radian into the meter unit, the phase to elevation operator should be applied. The acquisition geometry for both images (master and slave), phase noise and possible unwrapping errors significantly affect this step [57].

TC will geocode the final bands by correcting geometric distortions of SAR images using a ready to download DEM and, lastly, produce a projected map. On the other hand, TC (geocoding) converts an image from either slant range (SR) or ground range (GR) into a coordinate system. Finally, this step using a DEM corrects geometric distortions of foreshortening, layover, and shadow.

### 3.3. Validation and Comparison

#### 3.3.1. Standard Errors of the Estimate

Generally, the accuracy assessment is vital in approving the quality of extracted information from remotely sensed data [60]. Regression analysis is associated with a collection of statistical techniques, where the behavior of random variables (dependent variables) using quantitative variables (independent variables) are described [61,62]. The correlation coefficient (R) has the values between +1 and −1, where 1 determines a total positive linear correlation, 0 represents no linear correlation, and −1 shows the total negative linear correlation [63,64]. The accuracy of predictions is measured by the standard error of the estimate and defined by the following equation [65,66]:

(4) $$\sigma_{est}=\sqrt{\frac{\sum (\overset{}{\overset{\wedge }{Y}-Y{)}^{2}}}{N-2}}$$

where, $\sigma_{est}$ is the standard error of the estimate,$\;\hat{Y}$ is estimate variables, *Y* is an actual variable, and *N* is the number of GCPs. The numerator is squared differences between the actual variables and the estimated one. As a whole, the lower the numerator, the higher the accuracy will be [65,66].

#### 3.3.2. Hydrological Delineation

By delineating the stream networks for the created DEM from Sentinel-1 and the other products of AIRSAR, TanDEM-X, ALOS-PALSAR and SRTM in the GIS environment using the hydrology toolbox, the study was well-validated and justified as well. Based on the quality of the decorrelation of phase and spatial resolution for each DEM, many stream orders can be extracted in this method. The higher the spatial resolution, the more the stream orders can be delineated. Based on the system’s structure, rivers of the order number “1” are the highest tributaries. Thus, if two streams of the outermost tributaries merge, it gives a higher order. Therefore, whenever two streams with different orders merge, it gives a higher-order number than them.

The DEMs were used to delineate streams one by one. For example, the created DEM from Sentinel-1 was imported into GIS, and a hydrology toolbox was employed for extracting stream orders for it. In the first step, the filling operator was utilized for filling any possible gap in the DEM. In the second step, the flow direction operator was applied for obtaining all directions of water flow in the DEM. At the third stage, using the flow accumulation raster threshold, those in the same direction or path were connected. Next, by using a stream link, the streams in the same directions were linked together. Then, a stream order operator was applied to order the streams from 1 to whatever. Finally, the extracted rivers were converted to feature using stream to feature operator. It is worth mentioning that these processes were applied for all the DEMs.

#### 3.3.3. Validation Using Ground Control Points (GCPs)

The vertical accuracy of the DEMs was validated via comparing the interpolated elevations from each DEM and global navigation satellite system (GNSS) ground control points (GCPs). All DEMs of the both study areas were validated through available GCPs. The static GCPs (validation points) were measured using a GPS (geodetic receivers). Trimble device in Malaysia and Leica device in Iran were placed for minimum three hours until the signals were received from the satellites (based on the nearest continuously operating reference stations). The horizontal and vertical accuracy of each gcpare not exceeded ±2 cm and ±5 cm, respectively.

Available eight GCPs for Malaysia and six for Iran were used for validation. Normally, available GCPs are rare for example, Halim, et al. [67] used 45 GCPs (in 198,000 km^2^ meaning 1 GCP per 4400 km^2^) for a study performed in East of Malaysia. And in another study done in Perlis region in Malaysia, Pa’suya, et al. [68], used 38 GCPs (in 810 km^2^ meaning 1 GCP per 21 km^2^). The obtained elevations from DEMs compared with the corresponding elevation values of the GCPs. The outlier test using interquartile range (*IQR*) was done for the discrepancy between each DEM and the GCPs based on Equation 5 [69].

(5) Outlier=(Q3−Q1)+1.5 IQR

where, *IQR* is Interquartile range, *Q*1 represents the lower quartile, and *Q*3 is the upper quartile.

Accordingly, the Root Mean Square Error (RMSE) for the discrepancy between each DEM and the GCPs were also computed using Equation 6 [70].

(6) $$RMSE=\sqrt{\frac{\sum_{i=1}^{n}{\left[X\left(predicted\right)-X\left(measured\right)\right]}^{2}}{n-1}}$$

Where, X(measured) indicates the elevation of the GCPs, X(predicted), defines the elevation extracted from the DEMs, and n is the total number of GCPs.

## 4. Results

### 4.1. DEM Creation from Sentinel-1 Using InSAR Technique

**Figure 4:**
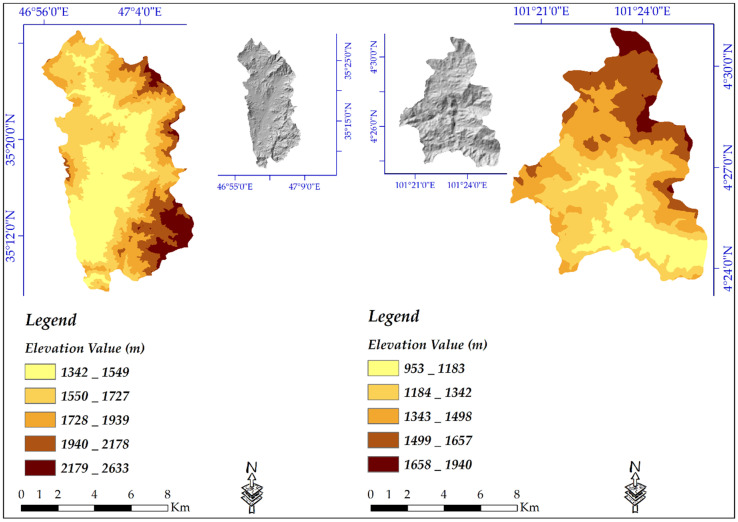
Extracted DEM from Sentinel_1 Satellite Data.4.2. Evaluation and Comparison of the Created DEM.

Using InSAR technique, for each study area, a DEM was extracted from Sentinel-1 data. Because of low coherence, vegetated areas typically will produce poor elevation value in C-band [44,71]. Our results also showed that DEM from L-band (ALOSPALSAR) is more suitable than DEM from C- band (Sentinel-1). Because of the inherent characteristics of ALOS PALSAR L-band data, the accuracy of its resulted DEM is normally better than the Sentinel-1 and TanDEM-X DEMs. However, in Iran’s selected study area, mostly covered by rangelands, the DEM accuracy was also slightly better than Malaysia’s study site, where forests are dominant. Figure 4 shows the created DEM from Sentinel-1 for both areas.

#### 4.1.1. The Linear Regression and Standard Errors of the Estimate

DEMs vary in quality and pixel sizes depending on the creation methods and data used [72]. The linear regression as one of the reliable ways to validate raster layers [73] was used to evaluate the relative accuracy of the created DEM. The extracted DEM from Sentinel-1 imagery was 14-m cell size. Therefore, a 12.5 × 12.5 spatial resolution DEM of the ALOS-PALSAR products was selected as the reference for validating using linear regression.

**Figure 5:**
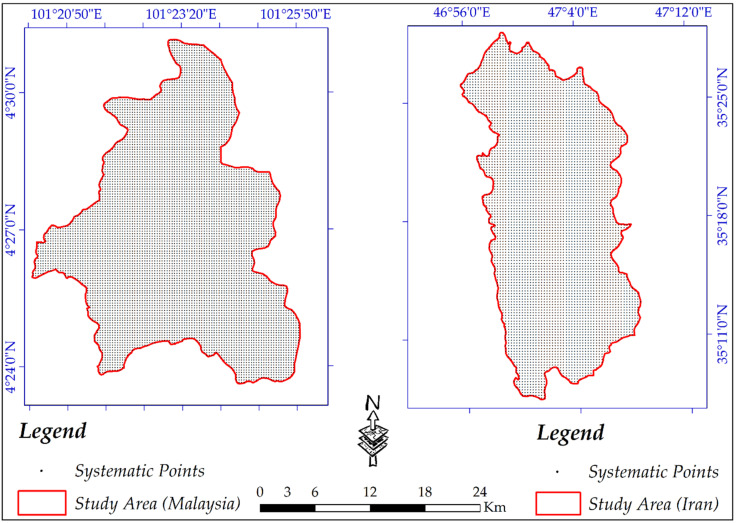
The location of the *systematic* points.

**Figure 6:**
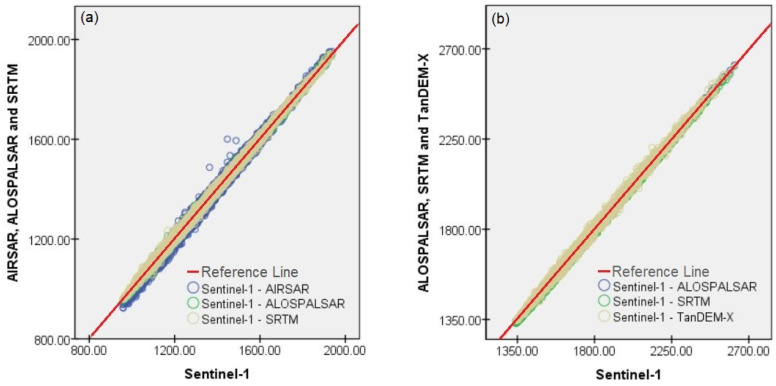
Scatter plot; (**a**) Malaysia and (**b**) Iran.

The linear regression and standard errors of the estimate were employed to validate the extracted DEM’s relative accuracy. For this reason, 8120 and 5334 systematic points for Malaysia and Iran were selected, respectively (Figure 5) were created and the elevation values from DEMs were exported into them. These points were employed to validate (relative accuracy) the DEMs using the linear regression (Table 2) and scatter plots (Figure 6) for the both areas. The correlation coefficient was 99% and 100% for 2 study areas of Malaysia and Iran, respectively. This fact confirms that the variables were fully correlated (positive linear correlation). The estimated standard error was quite high for either study area, but for the Cameron Highlands, this was more noticeable (almost 7 m); because on one hand, vegetation coverages mostly covers the study area and C-band SAR data cannot penetrate dense vegetation coverages, on the other hand, the short PB can be another reason. As it can be seen from the scatter plot, for the study area of Iran, where is mostly covered by spare vegetation coverages, the values are more correlated than the denser vegetated area of Malaysia.

#### 4.1.2. Validation Using GCPs

**Figure 7:**
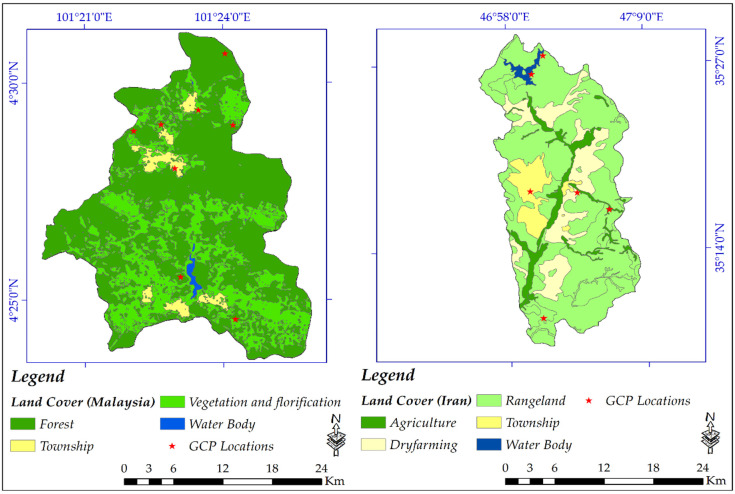
Geographical locations of the Ground Control Points (GCPs) on the land cover maps.

To assess the results’ quality and validity, several available GCPs points were collected to be compared with all DEMs including the created DEM from Sentinel-1 SAR satellite imagery. Figure 7 shows eight GCPs for Malaysia and six GCPs for Iran over land cover type map.

The results showed differences between them, meaning that based on the GCP points, the DEM created from Sentinel-1 could not be reliable. Therefore, the researchers should be cautious about using it as a base DEM for their studies. The outlier test results show that there are no outlier concerning the obtaining elevation values from the DEMs for both study areas (Table 3).

In fact, establishing GCPs is a time-consuming, hard job, costly, and even impossible sometimes [74]. The GCPs are essential for validating a DEM based on their actual elevations using Root Mean Square Error (RMSE) [72,75]. The validation technique using GCPs was earlier employed by the United States and Japanese researchers for the accuracy assessment of the Advanced Space-borne Thermal Emission and Reflection Radiometer (ASTER) [76]. Results indicate that the ALOSPALSAR for Iran and AIRSAR for Malaysia were the best DEMs with total RMSE of ±5.2 m and ±6.4 m, respectively (Table 4 and Table 5).

Accuracy assessment using the available GCPs was performed for the created DEM from Sentinel-1 based on the land cover types (Table 6). The interferometric measurements is normally affected by land covers [77]. To assess the impacts of different land covers on the accuracy of the created DEM using Sentinel-1, the GCP points were grouped based on the corresponding land cover types. In the study area of Malaysia, GCPs number 2, 4, and 8 were located in vegetation and florification, GCPs number 1, 3, 5, and 7 were lies on forests and GCP number 6 was located on township. At the same time, in the study area of Iran, GCPs number 1, 5, and 6 were placed in rangeland, and GCPs number 2, 3, and 4 were located in township, agriculture and dryfarming, respectively.

Overall, RMSE values are quite high and ranges from five to thirteen meters in the study area of Malaysia, were mostly covered by dense forest and vegetation. In Malaysia, the highest RMSE of 13 m can be seen in township (this maybe because there are high rising hotels in the Cameron Highlands with sparse trees coverages). Then, the highest value was calculated for forests of 11.7 m, followed by vegetation and florification with 5.6 m. Because of low vegetation coverages these values were quite lower in Iran than Malaysia, but because of the technical issues the RMSE for Iran also showing that Sentinel-1 failed to create an accurate DEM. In Iran, sparse and bare lands are contributed to lower RMSE meaning that agriculture, dryfarming, and rangeland showed lower RMSE of 7, 5, and 3.5 m, respectively. The township has RMSE of 9 m.

#### 4.1.3. Hydrological Analysis

Hydrological units are used for the planning and management of natural resources. To better understand the low accuracy of the extracted DEM, the hydrological analysis was performed as well. Therefore, the created DEMs were hydrologically compared with AIRSAR, ALOS-PALSAR. TanDEM-X, and SRTM products. The hydrological networks were delineated for all four DEMs using the hydrology toolbox in the GIS environment. The concern with the extracting stream networks, given the same accumulation raster layer, the higher threshold value contributes to less dense stream orders and vice versa [78].

**Figure 8:**
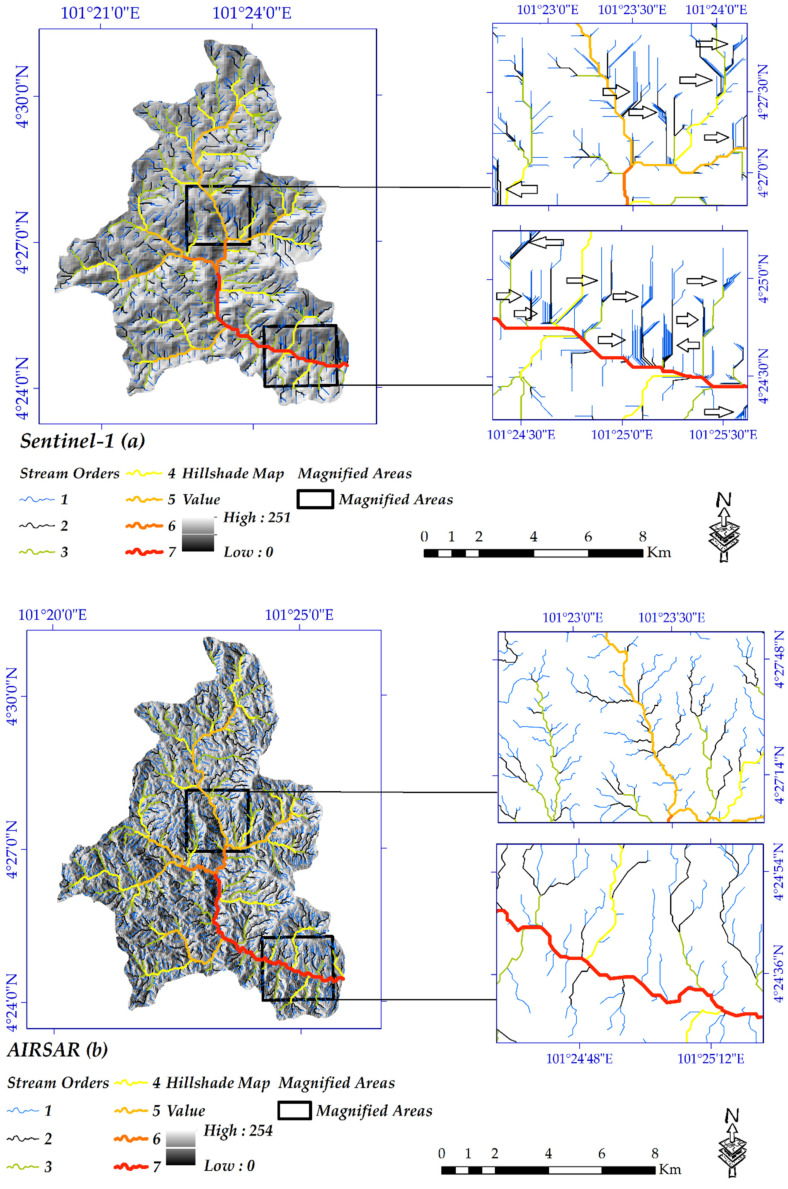

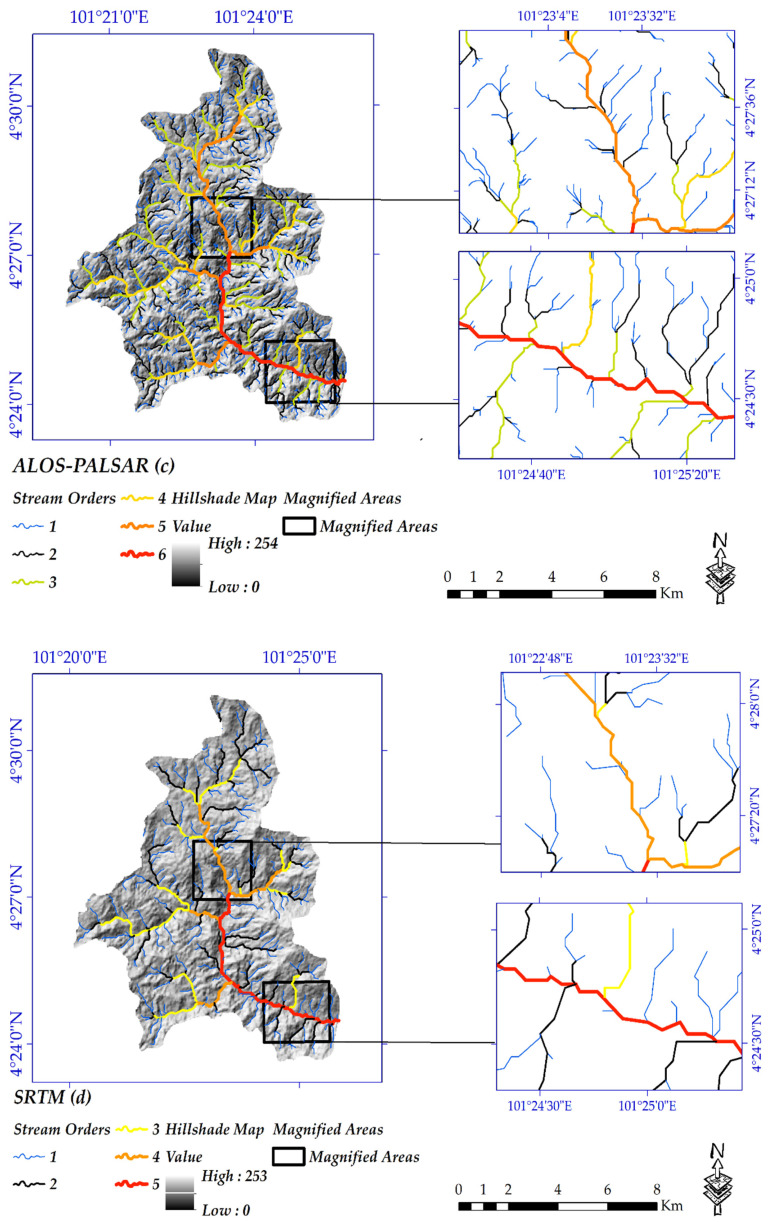
DEMs of (**a**) Sentinel-1; (**b**) airborne synthetic aperture Radar (AIRSAR); (**c**) advanced land observing satellite phase array L-band synthetic aperture radar (ALOS-PALSAR); and (**d**) SRTM.

**Figure 9:**
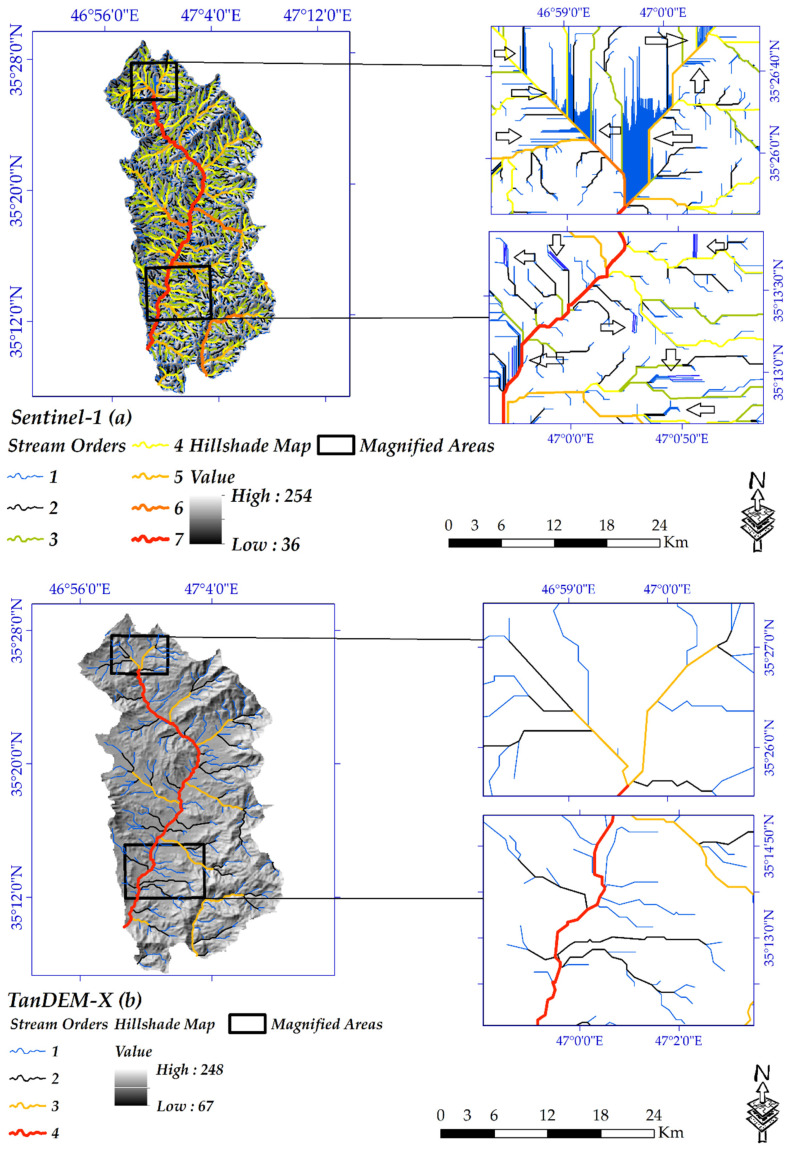

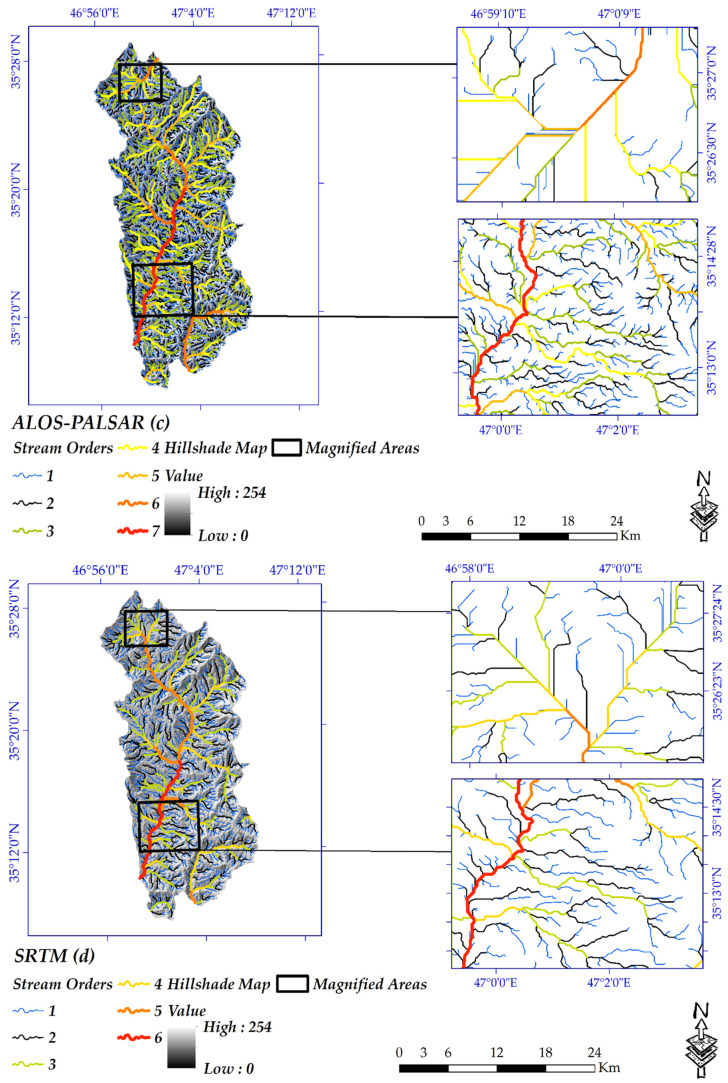
DEMs of (**a**) Sentinel-1; (**b**) TanDEM-X; (**c**) ALOS-PALSAR; and (**d**) SRTM.

Figure 8 and Figure 9 clearly show the differences between the extracted DEM from Sentinel-1 data with the other DEMs for both study areas. Because of the close spatial resolution and the same accumulation threshold in GIS, the equal number of stream orders was delineated for extracted DEM from Sentinel-1 data and the ALOS-PALSAR in either study area. Moreover, because of the higher spatial resolution, AIRSAR DEM contributes to the highest stream orders, While, regarding the lower spatial resolution than the other, DEM of SRTM has only five stream orders in the Cameron Highlands. Regarding Iran’s study area, because of the lower spatial resolution, the lower stream orders were extracted from TanDEM-X and SRTM. The wavelength, PB and TB have an essential role in the accuracy of a DEM. More importantly, because of poor coherence and interferogram resulted from the low PB and short wavelength, the extracted DEMs from Sentinel-1 (C-band) showed too many noises in-stream orders, exceptionally orders number one (Figure 8a and Figure 9a). Simultaneously, such noises cannot be seen in the other DEMs, even DEM of SRTM and TanDEM-X with the lower cell size.

## 5. Discussion

### 5.1. Limitations of InSAR Technique

The nature of the InSAR technique is noisy and this approach is limited to coarse scene descriptions. At the same time, in built-up areas it result in layover and shadow [79]. InSAR limits depends on the spatial extent and magnitude of features, which depends upon geometric attributes of data used (L-band or C-band) [79,80]. Although it is technically possible to create an interferogram, InSAR is not an ideal technique for dense vegetated areas [80], especially when the images are also geometrically unusable for such areas. To obtain better results from InSAR technique, it is better to use the longer wavelength images such as L-band and more importantly using it in less vegetated areas.

#### Multi-Temporal InSAR and PSInSAR Approaches in DEM Generation over Vegetated Areas

For such areas like the Cameron Highlands (Malaysia), where mostly covered by dense vegetation coverages, multi-temporal InSAR technique, which integrates different estimators, resulting in a self-adaptive and reliable data processing [81,82]. Another advantage of this model is that, it is self-adaptive and the algorithm works well under various situations (more accurate coherence estimation); therefore, it offers more promising results than InSAR for surface deformation studies and DEM creation [81,83]. Another technique that can be used for DEM generation in vegetated areas is the Permanent scatterer (PSInSAR), which is different completely from the traditional InSAR that firstly developed by Ferretti, et al. [84]. The results of this model is by far more accurate than InSAR [85,86]. For reliable results, unlike InSAR, this technique requires many interferometric data pairs (more than 20, the more the data the more the result will be accurate) [86]. The PSInSAR calculates the motion of scatterers and the propagation delay due to changes [86,87]. This technique has been proved effective in many surface deformation-monitoring studies [87].

### 5.2. Atmospheric Delays

Short temporal baseline, suitable perpendicular baseline, and suitable atmospheric conditions for data acquisition are very essential and should be considered [46,88,89]. In this study, all above conditions were considered carefully. For example, water vapor in the atmosphere result in phase delays and decreases the quality of the measurement [46,88]. Therefore, it is advisable to collect images obtained during dry days and also during no rainfall days. Hanssen [89], suggested an alternative for this and mentioned that try to collect nighttime’s data, which are normally less affected by atmospheric conditions.

Due to the good temporal characteristics of the data used in this study, we did not apply atmospheric delays correction. Date of the data used for the study area (Malaysia) are in months of late February and early March. In addition, for the study area (Iran) the data are in months of late July and mid-August. Refer to the weather condition in the study areas, we can find out that the images were not much affected by hot days (water vapor) and rainfall (wet days). Because there are two main monsoon seasons in Malaysia from late May to September and from October to February [90]. In Iran also the data were collected during the times that neither there is rainfall nor very hot days [91].

Although, based on the above-mentioned statements, this paper did not used any method for removing atmospheric path delays, but suggests a few models to be used in the future studies (especially, where researchers cannot collect suitable data), including direct observations of atmospheric properties (e.g., continuous GPS observations), empirical corrections [92], weather models [93], global atmospheric models [94], ERA5 global atmospheric model [95], and MERIS water vapor data and elevation-dependent interpolation model [96]. Atmospheric delays can be classified as neutral and ionospheric components, which ionospheric component is associated with the free electrons in the atmosphere [92,97]. The neutral atmospheric component is usually caused by a combination of non-dipole (hydrostatic) and dipole (water vapor) components [92,97]. The non-dipole delay dominates with delays of almost 2 m, while the dipole is associated with approximately a few tens of centimeters [97].

### 5.3. Effects of Different Wavelengths on DEM Products

The X-, C- and L-band SAR signals have a different interaction with surface objects. Typically, X-band signals backscatter from the top of the trees canopy, while C-band signals penetrate deeper into the canopy [98]. In contrast, L-band signals provide a greater penetration rate than X- and C-bands [71,99,100,101]. The estimated interferometric phase and the coherence between two images (master and slave) is generally an indicator for assessing the phase information quality, which have direct influences on DEM generation. The coherence value ranges from 0–1. The more the value the better the quality of the interferogram will be [44,102]. The different wavelengths have a different interaction with different land covers [44,102,103]. For example, the coherence coefficient calculated from L-band ALOSPALSAR is normally higher than C-band Sentinel-1 and X-band TanDEM-X in vegetated areas [44]. When SAR signals could not penetrate vegetation, then coherence will be poor (dark) [104]. Therefore, coherence loss causes poor interferometric results [45,71], which result in a low quality DEM. Short wavelength SAR sensors of C- and X-bands (because of low penetration rate) have poor coherence (dark) in vegetation areas (this differs based on the density of vegetation); while having a slightly higher coherence (bright) in township areas. Compared to the C- and X-bands that become uncorrelated quickly, because of a better coherence, L-band interferometry is more suitable for denser vegetated areas [98,103]. The interferogram is generally shown in a color scale that ranges from -π to +π (also, called “fringes” and it is displayed as a set of arbitrary colors’ cycles) [45]. The dense interferometric fringe is generated by X-band, while the relatively sparse fringes are derived from Sentinel-1 and ALOSPALSAR in order [44]. To create a high quality DEM, these fringes should be present throughout the image [45]. Like the wavelength, the land cover also affects the DEM quality [41,44]. Accordingly, phase information (interferogram) over vegetated areas, which have low coherence will not produce accurate elevation measures. For the denser vegetated coverage areas, it is better to use L-band satellite data to create a DEM rather than C and X-bands [67,103].

#### Co-Registration of Different DEMs Using the Least Trimmed Squares (LTS) Estimator

Different DEMs only with common coordinate systems can be compared [105,106,107]. In this study, the least trimmed squares (LTS) estimator was used to co-register the DEMs, which was also applied by Zhang and Cen [105]. The model is a simple but high-performance technique to co-register the different DEMs, which was initially introduced by [108]. The algorithm does not need any prior information about the terrain changes [105]. In addition, identifying the terrain changes with a self-adaptive threshold and very high matching and change detection accuracy are among advantages of the model [105].

The optimum perpendicular baseline (PB) for DEM creation should be between 150 to 300 m [45,46]. The higher and the lower PB can cause loss of coherence and leading to decorrelation (due to high sensitivity to phase noise and atmospheric effects). Simultaneously, the temporal baseline (TB) should be short and usually 6 to 12 days at best [46]. The Sentinel-1 mission was basically designed for the deformation studies not for DEM generation [100]. Therefore, PB above 100 m are hard to find [45]. Most of the image pairs have a baseline below 30 m and pairs with short TB and large PB can be hard to find [45,100]. In fact, this is the main reason why it cannot be applied for DEM generation. However, because of the abovementioned statements and knowing that Sentinel-1 is a C-band SAR data, it cannot be employed for DEM generation in vegetated areas. Accordingly, because of the low PB, creating DEM from Sentinel-1 over bare lands will also produce bad results.

In terms of data acquisition and generation processes, creating a DEM using SAR data is a time-consuming issue [109]. It is worth mentioning that if a researcher can collect data with the optimum PB and TB; the best spatial resolution for Sentinel-1 will be around 13 m [110]. The quality of the DEM generation is strongly affected by atmospheric disturbances and coherence. From Sentinel-1, a PB higher than 100 m is hard to find. Therefore, creating the DEM is a challenging issue. Despite the higher spatial resolution, the short PB and wavelength result in errors, so the created DEM has not better quality than of freely available DEMs, including SRTM.

Based on findings using evaluation techniques of the linear regression, ground control points and hydrological models, the created DEM has low accuracy. The linear regression showed more error for dense vegetated areas. Moreover, the hydrological networks for the created DEM, showed bad results, especially in stream orders number one. Additionally, results by GCPs were also confirmed that creating DEM from Sentinel-1 data is not giving promising result, especially over the vegetated areas.

## 6. Conclusions

It is noted that DEM generation due to numerous usages for extracting different attributes, such as slope, aspect, elevation, curvature, etc., is one of the essential spatial information tools used in the earth sciences. Therefore, optical and radar satellite images are the two main sources to produce DEM. DEM generation with the SAR interferometry approach is one of the most common methods and It is very interesting for geosciences researchers. Furthermore, one of the most recent Sentinel-1 missions is SAR imagery. Sentinal-1 images are among the best earth observations for DEM generation due to acquire images from the whole earth surface once every six-day, availabilities, high-resolution, and C-band sensitivity to the surface structure.

The objective of this study was to produce a DEM from Sentinel-1 satellite imagery using the InSAR technique along with to compare it with DEMs of AIRSAR, ALOS-PALSAR, TanDEM-X, and SRTM. For this reason, Sentinel-1 products for two different study areas were acquired, from which DEM with 14-m pixel size was obtained for a part of the Cameron Highlands, Pahang, Malaysia, and a part of Sanandaj, Iran. Based on the analysis and comparisons with the other proven DEMs using Linear regression and hydrological models, the quality of derived DEM for the two study areas was less than the other DEMs, even 90- and 30-m cell size DEM of TanDEM-X and SRTM, respectively. At the same time, results of RMSE using a few available GCPs showed that the created DEMs were not giving promising results. The validation results showed that Sentinel-1 could not produce accurate DEMs for two reasons. Firstly, the C-band signals cannot penetrate the denser vegetation coverage, especially canopies and trees. Secondly, the relatively low accuracy can be due to the small perpendicular baseline of Sentinel-1 data, which is rarely longer than 100 m could be found. For creating a DEM, the baseline should generally be between 150 to 300 m. This study recommends researchers to use freely available DEMs (e.g., SRTM) instead of creating DEM from Sentinel-1. Finally, if a DEM with a higher spatial resolution is required, they can use other products such as ALOS-PALSAR and LiDAR (Light Detection and Ranging) data, which is a technique to measure distances by target illuminating using a laser and calculating the backscatters with a sensor.

## Figures and Tables

**Table 1:** Technical characteristics of Sentinel-1 were used in this study.

Input Product		PB (m)	TB (day)	Sub-Swath	Polarization	Spatial Resolution (m)
Malaysia	S1A_IW_SLC_20170220 S1A_IW_SLC_20170304	47	12	IW1	VV	5 × 20
Iran	S1B_IW_SLC_20200816 S1B_IW_SLC_20200723	109	25	IW1	VV	5 × 20

**Table 2:** The values of linear regression and standard errors of the estimate.

Statistical Parameters Study Area	R (%)	Std. Error of the Estimate (m)
Malaysia	99	6.87
Iran	100	4.31

**Table 3:** Minimum and maximum outlier values for the both study areas.

No.	Study Area (Iran)			Study Area (Malaysia)		
	DEMs	Minimum Outlier Value (m)	Maximum Outlier Value (m)	DEMs	Minimum Outlier Value (m)	Maximum Outlier Value (m)
1	ALOSPALSAR	3.15	5.95	ALOSPALSAR	2	9.8
2	SRTM	–5.5	14.5	SRTM	–5.5	22.5
3	Sentinel-1	–6.5	15.5	Sentinel-1	–6.2	13
4	TanDEM-X	–13.3	36.4	AIRSAR	–2.4	13.4

**Table 4:** Validation of the Digital elevation model (DEM) using GCPs (Iran).

UTM Coordinates and Elevation of GCPs^1^.				Corresponding Elevation of the DEMs			
No.	X (m)	Y (m)	H (m)	ALOS (m)	SRTM (m)	Sentinel (m)	TanDEM-X (m)
1	680,712	3,908,740	1544	1548	1540	1551	1541
2	682,441	3,904,790	1436	1441	1427	1439	1441
3	689,659	3,908,010	1598	1604	1591	1608	1612
4	678,987	3,923,820	1555	1551	1550	1561	1562
5	678,196	3,925,250	1588	1593	1586	1592	1606
6	682,564	3,890,380	1553	1558	1551	1549	1572
RMSE (m)				±5.2	±6.0	±6.7	±13.7
1 UTM = Universe Transverse Mercator Projection.							

**Table 5:** Validation of the DEMs using GCPs (Malaysia).

UTM Coordinates and Elevation of GCPs				Corresponding Elevation of the DEMs			
No.	X (m)	Y (m)	H (m)	ALOS (m)	Sentinel (m)	SRTM (m)	AIRSAR (m)
1	765,837	498,361	1611	1621	1622	1617	1615
2	763,839	496,053	1484	1489	1482	1479	1487
3	765,322	496,134	1554	1562	1562	1551	1561
4	764,306	496,469	1461	1466	1464	1466	1464
5	765,691	495,121	1546	1550	1559	1554	1554
6	768,850	493,748	1678	1682	1691	1688	1687
7	764,026	489,596	1190	1196	1196	1198	1186
8	768,510	488,383	1143	1149	1150	1148	1137
RMSE (m)				±6.8	±9.5	±7.1	±6.4

**Table 6:** The Root Mean Square Error (RMSE) of Sentinel-1 DEM based on the GCPs for the study areas.

GCP No.	Land Cover	Malaysia	GCP No.	Land Cover	Iran
		RMSE (m)			RMSE (m)
2	Vegetation and florification	5.6	1	Rangeland	3.5
4			5		
8			6		
1	Forest	11.4	4	Dryfarming	5.0
3			3	Agriculture	7.0
5					
7					
6	Township	13.0	2	Township	9.0

## References

[1] Ai T., Li J. (2010). A DEM generalization by minor valley branch detection and grid filling. ISPRS J. Photogramm. Remote Sens..

[2] Arun P.V. (2013). A comparative analysis of different DEM interpolation methods. Egypt. J. Remote Sens. Space Sci..

[3] Bhattacharya A., Arora M.K., Sharma M.L. (2012). Improved digital elevation model creation using SAR interferometry in plane and undulating terrains. Himal. Geol..

[4] Mokarrama M., Hojati M. (2016). Landform classification using a sub-pixel spatial attraction model to increase spatial resolution of digital elevation model (DEM). Egypt. J. Remote Sens. Space Sci..

[5] Chappell A., Heritage G.L., Fuller I.C., Large A.R., Milan D.J. (2003). Geostatistical analysis of ground-survey elevation data to elucidate spatial and temporal river channel change. Earth Surf. Process. Landf. J. Br. Geomorphol. Res. Group.

[6] Liu X., Zhang Z., Peterson J., Chandra S. (2007). LiDAR-derived high quality ground control information and DEM for image orthorectification. GeoInformatica.

[7] Eisenbeiß H. (2009). UAV Photogrammetry.

[8] Kervyn F. (2001). Modelling topography with SAR interferometry: Illustrations of a favourable and less favourable environment. Comput. Geosci..

[9] Perna S., Esposito C., Berardino P., Pauciullo A., Wimmer C., Lanari R. (2015). Phase offset calculation for airborne InSAR DEM generation without corner reflectors. IEEE Trans. Geosci. Remote Sens..

[10] Maghsoudi M., Navidfar A., Mohammadi A. (2017). The sand dunes migration patterns in Mesr Erg region using satellite imagery analysis and wind data. Nat. Environ. Chang..

[11] Mohammadi A., Kamran K.V., Karimzadeh S., Shahabi H., Al-Ansari N. (2020). Flood Detection and Susceptibility Mapping Using Sentinel-1 Time Series, Alternating Decision Trees, and Bag-ADTree Models. Complexity.

[12] Potin P., Bargellini P., Laur H., Rosich B., Schmuck S. Sentinel-1 mission operations concept. Proceedings of the Geoscience and Remote Sensing Symposium (IGARSS), 2012 IEEE International.

[13] Mohammadi A., Baharin B., Shahabi H. (2019). Land Cover Mapping Using a Novel Combination Model of Satellite Imageries: Case Study of a Part of the Cameron Highlands, Pahang, Malaysia. Appl. Ecol. Environ. Res..

[14] Mohammadi A., Bin Ahmad B., Shahabi H. (2018). Extracting digital elevation model (dem) from sentinel-1 satellite imagery: Case study a part of cameron highlands, pahang, Malaysia. Int. J. Manag. Appl. Sci.

[15] Taud H., Parrot J.-F., Alvarez R. (1999). DEM generation by contour line dilation. Comput. Geosci..

[16] Escobar Villanueva J.R., Iglesias Martínez L., Pérez Montiel J.I. (2019). DEM generation from fixed-wing UAV imaging and LiDAR-derived ground control points for flood estimations. Sensors.

[17] Zietara A.M. (2017). Creating Digital Elevation Model (DEM) Based on Ground Points Extracted from Classified Aerial images Obtained from Unmanned Aerial Vehicle (UAV).

[18] Zhou P., Tang X., Guo L., Wang X., Fan W. (2018). DEM generation using Ziyuan-3 mapping satellite imagery without ground control points. Int. J. Remote Sens..

[19] Höhle J. (2009). DEM generation using a digital large format frame camera. Photogramm. Eng. Remote Sens..

[20] Lee C., Jones S., Bellman C., Buxton L. (2008). DEM creation of a snow covered surface using digital aerial photography. Int. Arch. Photogramm. Remote Sens. Spat. Inf. Sci..

[21] Gooch M.J., Chandler J.H., Stojic M. (1999). Accuracy assessment of digital elevation models generated using the Erdas Imagine OrthoMAX digital photogrammetric system. Photogramm. Rec..

[22] Henry J.B., Malet J.P., Maquaire O., Grussenmeyer P. (2002). The use of small-format and low-altitude aerial photos for the realization of high-resolution DEMs in mountainous areas: Application to the Super-Sauze earthflow (Alpes-de-Haute-Provence, France). Earth Surf. Process. Landf..

[23] Fabris M., Pesci A. (2005). Automated DEM extraction in digital aerial photogrammetry: Precisions and validation for mass movement monitoring. Ann. Geophys..

[24] Szypuła B. (2019). Quality assessment of DEM derived from topographic maps for geomorphometric purposes. Open Geosci..

[25] Ouerghi S., ELsheikh R.F.A., Achour H., Bouazi S. (2015). Evaluation and validation of recent freely-available ASTER-GDEM V. 2, SRTM V. 4.1 and the DEM derived from topographical map over SW Grombalia (test area) in North East of Tunisia. J. Geogr. Inf. Syst..

[26] Wehr A., Lohr U. (1999). Airborne laser scanning—an introduction and overview. ISPRS J. Photogramm. Remote Sens..

[27] Kucera L. (1999). Using ERS SAR Interferometry for DEM Creation in the Czech Republic.

[28] Takaku J., Futamura N., Goto A., Iijima T., Tadono T., Matsuoka M., Shimada M., Shibasaki R. High resolution DEM generation from ALOS PRISM data. Proceedings of the Geoscience and Remote Sensing Symposium.

[29] D’Ozouville N., Deffontaines B., Benveniste J., Wegmüller U., Violette S., De Marsily G. (2008). DEM generation using ASAR (ENVISAT) for addressing the lack of freshwater ecosystems management, Santa Cruz Island, Galapagos. Remote Sens. Environ..

[30] Sannidhi K., Kurian M. (2011). Creation of Digital Elevated Model using lunar images of Chandrayan-1. Int. J. Electr. Comput. Eng..

[31] Rossi C., Gernhardt S. (2013). Urban DEM generation, analysis and enhancements using TanDEM-X. ISPRS J. Photogramm. Remote Sens..

[32] Wang X., Holland D.M., Gudmundsson G.H. (2018). Accurate coastal DEM generation by merging ASTER GDEM and ICESat/GLAS data over Mertz Glacier, Antarctica. Remote Sens. Environ..

[33] Zink M., Bachmann M., Brautigam B., Fritz T., Hajnsek I., Moreira A., Wessel B., Krieger G. (2014). TanDEM-X: The new global DEM takes shape. IEEE Geosci. Remote Sens. Mag..

[34] Deng F., Rodgers M., Xie S., Dixon T.H., Charbonnier S., Gallant E.A., Vélez C.M.L., Ordoñez M., Malservisi R., Voss N.K. (2019). High-resolution DEM generation from spaceborne and terrestrial remote sensing data for improved volcano hazard assessment—A case study at Nevado del Ruiz, Colombia. Remote Sens. Environ..

[35] Fattahi H., Amelung F. (2013). DEM error correction in InSAR time series. IEEE Trans. Geosci. Remote Sens..

[36] Chang H.-C., Ge L., Rizos C., Milne T. Validation of DEMs derived from radar interferometry, airborne laser scanning and photogrammetry by using GPS-RTK. Proceedings of the Geoscience and Remote Sensing Symposium.

[37] Crosetto M. (2002). Calibration and validation of SAR interferometry for DEM generation. ISPRS J. Photogramm. Remote Sens..

[38] Shabou A., Baselice F., Ferraioli G. (2012). Urban digital elevation model reconstruction using very high resolution multichannel InSAR data. IEEE Trans. Geosci. Remote Sens..

[39] Baier G., Rossi C., Lachaise M., Zhu X.X., Bamler R. (2018). A Nonlocal InSAR Filter for High-Resolution DEM Generation from TanDEM-X Interferograms. IEEE Trans. Geosci. Remote Sens..

[40] Geymen A. (2014). Digital elevation model (DEM) generation using the SAR interferometry technique. Arab. J. Geosci..

[41] Gao X., Liu Y., Li T., Wu D. (2017). High Precision DEM Generation Algorithm Based on InSAR Multi-Look Iteration. Remote Sens..

[42] Faherty D., Schumann G.J.-P., Moller D.K. (2020). Bare Earth DEM Generation for Large Floodplains Using Image Classification in High-Resolution Single-Pass InSAR. Front. Earth Sci..

[43] Tian W., Zhao Z., Hu C., Wang J., Zeng T. (2019). GB-InSAR-based DEM generation method and precision analysis. Remote Sens..

[44] Fu B., Li Y., Gao E., Fan D., Lou P. (2020). Study on Accuracy Assessment of dem in the Marsh Using with Interferometric Palsar, SENTINEL-1A and Terrasar-X Images. Int. Arch. Photogramm. Remote Sens. Spat. Inf. Sci..

[45] Braun A. DEM Generation with Sentinel-1Workflow and Challenges. http://step.esa.int/docs/tutorials/S1TBX%20DEM%20generation%20with%20Sentinel-1%20IW%20Tutorial.pdf.

[46] ESA InSAR Processing: A Practical Approach. http://www.esa.int/esapub/tm/tm19/TM-19_ptB.pdf.

[47] Hutchinson C.S., Tan D.N.K. (2009). Geology of Peninsular Malaysia.

[48] Makoundi C., Zaw K., Large R.R., Meffre S., Lai C.-K., Hoe T.G. (2014). Geology, geochemistry and metallogenesis of the Selinsing gold deposit, central Malaysia. Gondwana Res..

[49] Mohammadi A., Shahabi H., Bin Ahmad B. (2018). Integration of Insar Technique, Google Earth Images and Extensive Field Survey for Landslide Inventory in a Part of Cameron Highlands, Pahang, Malaysia. Appl. Ecol. Environ. Res..

[50] Pradhan B., Sezer E.A., Gokceoglu G., Buchroithner M.F. (2010). Landslide susceptibility mapping by neuro-fuzzy approach in a landslide-prone area (Cameron Highlands, Malaysia). IEEE Trans. Geosci. Remote Sens..

[51] ESA SENTINEL-1 SAR User Guide Introduction. https://sentinel.esa.int/web/sentinel/user-guides/sentinel-1-sar.

[52] Geudtner D., Torres R., Snoeij P., Davidson M., Rommen B. Sentinel-1 system capabilities and applications. Proceedings of the Geoscience and Remote Sensing Symposium (IGARSS), 2014 IEEE International.

[53] Snoeij P., Attema E., Davidson M., Duesmann B., Floury N., Levrini G., Rommen B., Rosich B. The Sentinel-1 radar mission: Status and performance. Proceedings of the Radar Conference-Surveillance for a Safer World, 2009. RADAR. International.

[54] Massonnet D., Feigl K.L. (1998). Radar interferometry and its application to changes in the Earth‘s surface. Rev. Geophys..

[55] Yagüe-Martínez N., Prats-Iraola P., Gonzalez F.R., Brcic R., Shau R., Geudtner D., Eineder M., Bamler R. (2016). Interferometric processing of Sentinel-1 TOPS data. IEEE Trans. Geosci. Remote Sens..

[56] Luis V. TOPS Interferometry Tutorial. http://step.esa.int/docs/tutorials/S1TBX%20TOPSAR%20Interferometry%20with%20Sentinel-1%20Tutorial_v2.pdf.

[57] Rocca F., Prati C., Ferretti A. An Overview of SAR Interferometry. https://earth.esa.int/workshops/ers97/program-details/speeches/rocca-et-al/.

[58] Goldstein R.M., Werner C.L. (1998). Radar interferogram filtering for geophysical applications. Geophys. Res. Lett..

[59] Goldstein R.M., Zebker H.A., Werner C.L. (1988). Satellite radar interferometry: Two-dimensional phase unwrapping. Radio Sci..

[60] Alqurashi A.F., Kumar L. (2014). Land use and land cover change detection in the Saudi Arabian desert cities of Makkah and Al-Taif using satellite data. Adv. Remote Sens..

[61] Park S.H. (2011). Simple linear regression. International Encyclopedia of Statistical Science.

[62] Weisberg S. (2005). Simple linear regression. Applied Linear Regression.

[63] Pearson K. (1895). Note on regression and inheritance in the case of two parents. Proc. R. Soc. Lond..

[64] Stigler S.M. (1989). Francis Galton’s account of the invention of correlation. Stat. Sci..

[65] Chatterjee S., Hadi A.S. (2015). Regression Analysis by Example.

[66] Montgomery D.C., Peck E.A., Vining G.G. (2012). Introduction to Linear Regression Analysis.

[67] Halim S.M.A., Green M.F.P.s., Narashid R.H., Din A.H.M. Accuracy Assessment of TanDEM-X 90 m Digital Elevation Model In East of Malaysia Using GNSS/Levelling. Proceedings of the 2019 IEEE 10th Control and System Graduate Research Colloquium (ICSGRC).

[68] Pa’suya M.F., Din A.H.M., Amin Z.M., Omar K.M., Omar A.H., Rusli N. (2017). Evaluation of Global Digital Elevation Model for Flood Risk Management in Perlis.

[69] Gupta C.B., Gupta V. (2009). Introduction to Statistical Methods.

[70] Jalal S.J., Musa T.A., Ameen T.H., Din A.H.M., Aris W.A.W., Ebrahim J.M. (2020). Optimizing the Global Digital Elevation Models (GDEMs) and accuracy of derived DEMs from GPS points for Iraq’s mountainous areas. Geod. Geodyn..

[71] Wempen J.M., McCarter M.K. (2017). Comparison of L-band and X-band differential interferometric synthetic aperture radar for mine subsidence monitoring in central Utah. Int. J. Min. Sci. Technol..

[72] Wise S. (2007). Effect of differing DEM creation methods on the results from a hydrological model. Comput. Geosci..

[73] Mukherjee S., Joshi P., Mukherjee S., Ghosh A., Garg R., Mukhopadhyay A. (2013). Evaluation of vertical accuracy of open source Digital Elevation Model (DEM). Int. J. Appl. Earth Obs. Geoinf..

[74] Kaufmann V., Ladstädter R., Lieb G.K. Quantitative assessment of the creep process of Weissenkar rock glacier (Central Alps, Austria). Proceedings of the International Symposium on High Mountain Remote Sensing Cartography.

[75] Wise S. (2000). Assessing the quality for hydrological applications of digital elevation models derived from contours. Hydrol. Process..

[76] Team A.G.V. ASTER Global DEM Validation Summary Report. http://www.ersdac.or.jp/GDEM/E/3.html.

[77] Wessel B., Huber M., Wohlfart C., Marschalk U., Kosmann D., Roth A. (2018). Accuracy assessment of the global TanDEM-X Digital Elevation Model with GPS data. ISPRS J. Photogramm. Remote Sens..

[78] Chang K.-T. (2015). Introduction to Geographic Information Systems.

[79] Stilla U., Soergel U., Thoennessen U. (2003). Potential and limits of InSAR data for building reconstruction in built-up areas. ISPRS J. Photogramm. Remote Sens..

[80] Castellazzi P., Martel R., Galloway D.L., Longuevergne L., Rivera A. (2016). Assessing groundwater depletion and dynamics using GRACE and InSAR: Potential and limitations. Groundwater.

[81] Jiang M., Ding X., Li Z. (2013). Hybrid approach for unbiased coherence estimation for multitemporal InSAR. IEEE Trans. Geosci. Remote Sens..

[82] Zhao J., Wu J., Ding X., Wang M. (2017). Elevation extraction and deformation monitoring by multitemporal InSAR of Lupu Bridge in Shanghai. Remote Sens..

[83] Crosta G.B., Lollino G., Paolo F., Giordan D., Andrea T., Carlo R., Davide B. (2015). Rockslide monitoring through multi-temporal LiDAR DEM and TLS data analysis. Engineering Geology for Society and Territory-Volume 2.

[84] Ferretti A., Prati C., Rocca F. (2000). Nonlinear subsidence rate estimation using permanent scatterers in differential SAR interferometry. IEEE Trans. Geosci. Remote Sens..

[85] Tamburini A., Bianchi M., Giannico C., Novali F. (2010). Retrieving surface deformation by PSInSAR™ technology: A powerful tool in reservoir monitoring. Int. J. Greenh. Gas. Control..

[86] Kim J.-s., Kim D.-J., Kim S.-W., Won J.-S., Moon W.M. (2007). Monitoring of urban land surface subsidence using PSInSAR. Geosci. J..

[87] Cao N., Lee H., Jung H.C. (2015). A phase-decomposition-based PSInSAR processing method. IEEE Trans. Geosci. Remote Sens..

[88] Braun A. (2020). DEM Generation with Sentinel-1 Workflow and Challenges.

[89] Hanssen R.F. (2001). Radar Interferometry: Data Interpretation and Error Analysis.

[90] Lim J.T., Samah A.A. (2004). Weather and Climate of Malaysia.

[91] Amiri M., Eslamian S. (2010). Investigation of climate change in Iran. J. Environ. Sci. Technol..

[92] Fournier T., Pritchard M.E., Finnegan N. (2011). Accounting for atmospheric delays in InSAR data in a search for long-wavelength deformation in South America. IEEE Trans. Geosci. Remote Sens..

[93] Dong J., Zhang L., Liao M., Gong J. (2019). Improved correction of seasonal tropospheric delay in InSAR observations for landslide deformation monitoring. Remote Sens. Environ..

[94] Doin M.-P., Lasserre C., Peltzer G., Cavalié O., Doubre C. (2009). Corrections of stratified tropospheric delays in SAR interferometry: Validation with global atmospheric models. J. Appl. Geophys..

[95] Hu Z., Mallorquí J.J. (2019). An accurate method to correct atmospheric phase delay for insar with the era5 global atmospheric model. Remote Sens..

[96] Li Z., Xu W., Feng G., Hu J., Wang C., Ding X., Zhu J. (2012). Correcting atmospheric effects on InSAR with MERIS water vapour data and elevation-dependent interpolation model. Geophys. J. Int..

[97] Bevis M., Businger S., Herring T.A., Rocken C., Anthes R.A., Ware R.H. (1992). GPS meteorology: Remote sensing of atmospheric water vapor using the Global Positioning System. J. Geophys. Res. Atmos..

[98] Tanase M., de la Riva J., Santoro M., Pérez-Cabello F., Kasischke E. (2011). Sensitivity of SAR data to post-fire forest regrowth in Mediterranean and boreal forests. Remote Sens. Environ..

[99] Pope K.O., Rey-Benayas J.M., Paris J.F. (1994). Radar remote sensing of forest and wetland ecosystems in the Central American tropics. Remote Sens. Environ..

[100] Geudtner D., Prats P., Yague-Martinez N., Navas-Traver I., Barat I., Torres R. Sentinel-1 SAR Interferometry Performance Verification. Proceedings of the EUSAR 2016: 11th European Conference on Synthetic Aperture Radar.

[101] Ottinger M., Kuenzer C. (2020). Spaceborne L-Band Synthetic Aperture Radar Data for Geoscientific Analyses in Coastal Land Applications: A Review. Remote Sens..

[102] Lippl S., Vijay S., Braun M. (2018). Automatic delineation of debris-covered glaciers using InSAR coherence derived from X-, C-and L-band radar data: A case study of Yazgyl Glacier. J. Glaciol..

[103] Tang P., Zhou W., Tian B., Chen F., Li Z., Li G. (2017). Quantification of Temporal Decorrelation in X-, C-, and L-Band Interferometry for the Permafrost Region of the Qinghai–Tibet Plateau. IEEE Geosci. Remote Sens. Lett..

[104] Pichierri M., Hajnsek I., Zwieback S., Rabus B. (2018). On the potential of Polarimetric SAR Interferometry to characterize the biomass, moisture and structure of agricultural crops at L-, C-and X-Bands. Remote Sens. Environ..

[105] Zhang T., Cen M. (2008). Robust DEM co-registration method for terrain changes assessment using least trimmed squares estimator. Adv. Space Res..

[106] Breytenbach A., Van Niekerk A. (2020). Analysing DEM errors over an urban region across various scales with different elevation sources. South. Afr. Geogr. J..

[107] Jalal S.J., Musa T.A., Din A.H.M., Aris W.A.W., Shen W., Pa’suya M.F. (2019). Influencing factors on the accuracy of local geoid model. Geod. Geodyn..

[108] Rousseeuw P.J. (1984). Least median of squares regression. J. Am. Stat. Assoc..

[109] Reddy G.O., Kumar N., Sahu N., Singh S. (2017). Evaluation of automatic drainage extraction thresholds using ASTER GDEM and Cartosat-1 DEM: A case study from basaltic terrain of Central India. Egypt. J. Remote Sens. Space Sci..

[110] Forum S. DEM Resulted from Sentinel-1 Insar Are Very Similar to SRTM?. https://forum.step.esa.int/t/dem-resulted-from-sentinel-1-insar-are-very-similar-to-srtm/4492.

